# Supplementary material for: Risk Factors for Pressure Injuries in Adult Patients: A Narrative Synthesis
Source: Int J Environ Res Public Health. 2022 Jan 11;19(2):761. doi: 10.3390/ijerph19020761 (PMC8776011; doi:10.3390/ijerph19020761)
Supplement: Supplementary file 1 [file ijerph-19-00761-s001.zip › S2 Search Strategies.pdf]

Supplementary Material S2: search strategies

Search strategy PubMed		
#	1	"pressure ulcer*"[TIAB]
#	2	"pressure sore*"[TIAB]
#	3	"pressure injur*"[TIAB]
#	4	"pressure damage*"[TIAB]
#	5	"Pressure Ulcer"[mh]
#	6	"deep tissue injur*"[TIAB]
#	7	"skin ulcer*"[TIAB]
#	8	"bed sore*"[TIAB]
#	9	"superficial ulcer*"[TIAB]
#	10	decubitus[TIAB]
#	11	risk[TIAB]
#	12	„risk factors“[mh]
#	13	exposure[TIAB]
#	14	cause
#	15	predict*[TIAB]
#	16	prognos*[TIAB]
#	17	epidemiology[TIAB]
#	18	etiology[TIAB]
#	19	aetiology[TIAB]
#	20	extrinsic[TIAB]
#	21	external[TIAB]
#	22	intrinsic[TIAB]
#	23	internal[TIAB]
#	24	OR #1 - #10
#	25	OR #11 - #23
#	26	AND #24 - #25

Search strategy Embase		
#	1	"pressure ulcer*":ab,ti
#	2	"pressure sore*":ab,ti
#	3	"pressure injur*":ab,ti
#	4	"pressure damage*":ab,ti
#	5	"deep tissue injur*":ab,ti
#	6	"skin ulcer*":ab,ti
#	7	"bed sore*":ab,ti
#	8	"superficial ulcer*":ab,ti
#	9	decubitus:ab,ti
#	10	risk:ab,ti
#	11	„risk factors”:ab,ti
#	12	exposure:ab,ti
#	13	cause:ab,ti
#	14	predict*:ab,ti
#	15	prognos*:ab,ti
#	16	epidemiology:ab,ti
#	17	etiology:ab,ti
#	18	aetiology:ab,ti
#	19	extrinsic:ab,ti

#	20	external:ab,ti
#	21	intrinsic:ab,ti
#	22	internal:ab,ti
#	23	[humans]/lim
#	24	[embase]/lim
#	25	OR #1 - #9
#	26	OR #10 - #22
#	27	AND #23 - #26

**Search strategy Cochrane Library**		
#	1	pressure ulcer*
#	2	pressure sore*
#	3	pressure injur*
#	4	pressure damage*
#	5	deep tissue injur*
#	6	skin ulcer*
#	7	bed sore*
#	8	superficial ulcer*
#	9	decubitus
#	10	risk
#	11	risk factor
#	12	exposure
#	13	cause
#	14	predict*
#	15	prognos*
#	16	epidemiology
#	17	etiology
#	18	aetiology
#	19	extrinsic
#	20	external
#	21	intrinsic
#	22	internal
#	23	OR (#1 - #9):ti,ab,kw
#	24	OR (#10 - #22):ti,ab,kw
#	25	AND #23 - #24

**Search strategy Web of Science**		
#	1	pressure ulcer*
#	2	pressure sore*
#	3	pressure injur*
#	4	pressure damage*
#	5	deep tissue injur*
#	6	skin ulcer*
#	7	bed sore*
#	8	superficial ulcer*
#	9	decubitus
#	10	risk
#	11	risk factor
#	12	exposure

#	13	cause
#	14	predict*
#	15	prognos*
#	16	epidemiology
#	17	etiology
#	18	aetiology
#	19	extrinsic
#	20	external
#	21	intrinsic
#	22	internal
#	23	OR TS=(#1 - #9)
#	24	OR TS=(#10 - #22)
#	25	AND #23 - #24

**Search strategy CINAHL**		
#	1	MH “Pressure ulcer+”
#	2	TI pressure ulcer*
#	3	AB pressure ulcer*
#	4	TI pressure sore*
#	5	AB pressure sore*
#	6	TI pressure injur*
#	7	AB pressure injur*
#	8	TI pressure damage*
#	9	AB pressure damage*
#	10	TI deep tissue injur*
#	11	AB deep tissue injur*
#	12	TI skin ulcer*
#	13	AB skin ulcer*
#	14	TI bed sore*
#	15	AB bed sore*
#	16	TI superficial ulcer*
#	17	AB superficial ulcer*
#	18	TI decubitus
#	19	AB decubitus
#	20	TI risk
#	21	AB risk
#	22	TI risk factor
#	23	AB risk factor
#	24	TI exposure
#	25	AB exposure
#	26	TI cause
#	27	AB cause
#	28	TI predict*
#	29	AB predict*
#	30	TI prognos*
#	31	AB prognos*
#	32	TI epidemiology
#	33	AB epidemiology
#	34	TI etiology
#	35	AB etiology
#	36	TI aetiology
#	37	AB aetiology

#	38	TI extrinsic
#	39	AB extrinsic
#	40	TI external
#	41	AB external
#	42	TI intrinsic
#	43	AB intrinsic
#	44	TI internal
#	45	AB internal
#	46	OR #1 - 19
#	47	OR #20 - #45
#	25	AND #46 - #47
