# Supplementary material for: Risk Factors for Pressure Injuries in Adult Patients: A Narrative Synthesis
Source: Int J Environ Res Public Health. 2022 Jan 11;19(2):761. doi: 10.3390/ijerph19020761 (PMC8776011; doi:10.3390/ijerph19020761)
Supplement: Supplementary file 1 [file ijerph-19-00761-s001.zip › S3 Summary Table.pdf]

Supplementary Material S3: summary of studies

Table S1: Summary of studies included in narrative synthesis

Study	Study population	Design and analysis method	No. of people in final model (PI%), no. PI dev. and stage/grade outcome	No. of risk factors (no. of sig. factors), model risk factor names	p value	Odds ratio (OR)/Hazard Ratio (HR)	95% Confidence interval	Risk of bias
Aghazadeh et al. (2020)	200 pts<br>Setting: acute care hospital	Prospective cohort study<br><br>Logistic regression	200 (19.5%), 39<br>Stage ≥ 1	NR (3)<br><br>Total Braden score<br>Length of Hospitalization (days)<br>Weight (kg)	 <br>.03<br>.01<br>.01	OR<br><br>0.37<br>1.48<br>1.48	 <br>0.15-0.94<br>1.09-2.01<br>1.01-2.01	High
Allman et al. (1995)	286 pts.<br>Setting: tertiary care, urban, university teaching hospital	Prospective cohort study<br><br>Cox proportional hazard model	286 (12.9%), 37<br>Stage ≥ 2	9 (5)<br><br>Non-blanchable erythema of intact sacral skin<br>Lymphopenia<br>Immobility<br>Dry sacral skin<br>Decreased body weight	 <br>.05<br><br>.003<br>.02<br>.04<br>.03	HR<br><br>7.52<br><br>4.82<br>2.36<br>2.31<br>2.18	 <br>1.00-59.1<br><br>1.70-13.89<br>1.14-4.85<br>1.02-5.21<br>1.05-4.52	Moderate
Aloweni et al. (2019)	269 pts (surgical)<br>Setting: acute care hospital	Retrospective record review<br><br>Logistic regression	269 (28%), 80<br>Stage ≥ 1	13 (8)<br><br>Age ≥ 75 years<br>Female<br>ASA grade ≥ 3<br>BMI < 23<br>Preoperative Braden score ≤ 14<br>Anemia<br>Respiratory disease<br>Hypertension	 <br>NR<br>NR<br>NR<br>NR<br>NR<br>NR<br>NR<br>NR	OR<br><br>2.75<br>1.94<br>3.12<br>2.30<br>3.89<br>5.17<br>6.36<br>2.36	 <br>1.35-5.58<br>0.99-3.8<br>1.51-6.44<br>1.16-4.56<br>1.49-10.14<br>1.43-18.69<br>1.67-24.25<br>1.18-4.75	Moderate
Anrys et al. (2018)	308 pts<br>Setting: nursing home	Prospective cohort study<br><br>Logistic regression	308 (8.4%), 26<br>Stage ≥ 2	9 (3)<br><br>Pressure area-related pain<br>Non-blanchable erythema at baseline<br>Total Braden score	 <br>.038<br>.006<br>.031	OR<br><br>2.91<br>4.06<br>0.81	 <br>1.06-7.98<br>1.5-11.00<br>0.67-0.98	Moderate
Artico et al. (2020)	992 pts<br>Setting: residential hospice	Prospective cohort study	992 (26.5%), 263<br>Stage ≥ 1	16 (6)<br><br>Braden score (at risk vs. not at risk)<br>ESASr	 <br><.001	OR<br><br>3.14	 <br>1.87-5.27	Low

		Logistic regression		Feeling sleepy	.001	1.51	1.17-1.93	
				Pain	.007	1.48	1.17-3.14	
				Shortness of breath	.016	1.44	1.07-1.94	
				Primary diagnosis cancer (vs. other diagnoses)	.004	1.57	1.16-3.14	
				Urinary catheter	.015	1.48	1.08-2.03	
Baumgarten et al. (2004)	2285 pts Setting: nursing home	Prospective cohort study	1938 (23.3%), 450 Stage ≥ 2	14 (3)		HR		Moderate
				Black race	.032	1.31	1.02-1.66	
		Cox proportional hazard model		No. of activities of daily living dependencies	<.001	1.38	1.25-1.53	
				PI on admission	<.001	1.80	1.40-2.32	
Bergquist and Frantz (1999)	1711 pts Setting: urban home health care agency	Retrospective record review	1567 (3.5%), 55 Stage ≥ 2	45 (10)		HR		Moderate
				Limitation in activity to wheelchair	.02	2.77	1.18-6.51	
		Cox proportional hazard model		ADL dressing	<.001	2.71	1.54-4.78	
				Bowel/bladder incontinence	.02	2.85	1.18-6.84	
				Braden scale mobility	<.001	5.2	2.44-11.08	
				Anemia	.002	3.96	1.65-9.53	
				Adult child as primary caregiver	<.001	5.82	2.13-15.86	
				Male	.028	1.86	1.07-3.24	
				Recent fracture	.002	3.46	1.58-7.58	
				Oxygen use	<.001	3.95	2.06-7.57	
				Skin drainage	<.001	6.6	2.27-19.19	
Bergstrom and Braden (1992)	200 pts. Setting: nursing home	Prospective cohort study	200 (73.5%), 147 stage ≥ 1; (38.5%), 77 stage ≥ 2	*Model 1:* 10 (3)		OR[a]		Moderate
				Braden scale	<.01	0.69	NR	
		Logistic regression	Model 1: Stage ≥ 1	Diastolic blood pressure	<.01	0.95	NR	
				Protein (%RDA)	<.05	1.01	NR	
			Model 2: Stage ≥ 2					
				Model 2: 10 (3)				
			Model 3: Stage = 1					
				Braden scale	<.001	0.66	NR	
				Age	<.05	1.07	NR	
				Systolic blood pressure	<.01	0.97	NR	
				Model 3: 10 (4)				
				Braden scale	<.01	0.65	NR	
				Diastolic blood pressure	<.01	0.90	NR	
				Temperature (°F)	<.05	1.70	NR	
				Iron (%RDA)	<.01	0.98	NR	

Bergstrom et al. (1996)	843 pts. Setting: multiple	Prospective cohort study<br><br>Logistic regression	843 (12.8%), 108 Stage ≥ 1<br><br>Model 1: Preventive measures, age, gender, race, Braden scale<br><br>Model 2: Braden scale mobility and activity, primary diagnoses (13)<br><br>Model 3: Braden scale, primary diagnoses (13)	*Model 1:* 6 (3)		OR		Low
				Braden scale	<.001	1.30	1.19-1.41	
				Age	<.001	0.97	0.95-0.98	
				Race	.012	2.73	1.25-5.98	
				Model 2: 15 (3)				
				Braden scale mobility	<.001	1.72	1.27-2.33	
				Braden scale activity	.004	1.46	1.13-1.88	
				Cardiovascular Diagnosis	.023	2.49	1.14-5.48	
				Model 3: 14 (1)				
				Braden Scale	<.001	1.37	1.28-1.48	
Bourdel-Marchasson et al. (2000)	672 pts. Setting: hospital wards and geriatric units	RCT<br><br>Cox proportional hazard model	672 (44.5%), 299 Stage ≥ 1	NR (5)		HR		Moderate
				Control group vs. intervention group	.04	1.57	1.03-2.38	
				Norton score 5-10 vs. >14	.04	1.28	1.01-1.62	
				Kuntzmann score	.003	1.22	0.32-4.58	
				Serum albumin (1 g/L decrease)	<.001	1.05	1.02-1.07	
				Lower limb fracture	<.001	2.68	1.75-4.11	
Boyle and Green (2001)	534 pts. Setting: ICU	Prospective cohort study<br><br>Weibull regression	534 (5.2%), 28 Stage ≥ 1	2 (2)		HR		Moderate
				Coma/unresponsiveness/paralyzed & sedated	.001	4.2	30-77	
				Cardiovascular instability	.036	2.7	4-70	
Brandeis et al. (1994)	4232 pts. Setting: nursing homes	Prospective cohort study<br><br>Logistic regression	4232 (12.9%), 546 Stage ≥ 2<br><br>Model 1: high incidence homes 1322 (19.3%), 255<br><br>Model 2: low incidence homes 1365 (6.5%), 89	*Model 1:* 10 (4)		OR		Low
				Ambulation difficulty	<.001	3.3	2.0-5.3	
				Fecal incontinence	<.001	2.5	1.6-4.0	
				Diabetes mellitus	<.006	1.7	1.2-2.5	
				Feeding ADL	<.001	2.2	1.5-3.3	
				Model 2: 7 (3)				
				Ambulation difficulty	<.001	3.6	1.7-7.4	
				Feeding ADL	<.001	3.5	2.0-6.3	
				Male gender	<.001	1.9	1.2-3.6	
Chan et al. (2005)	666 pts. Setting: hospital wards	Prospective cohort study<br><br>Logistic regression	666 (8.1%), 51 Stage ≥ 1	23 (2)		OR		Moderate
				Braden scale (12-15) vs. Braden scale (16-23)	<.001	7.7	3.5-17.1	

				Braden scale (6-11) vs. Braden scale (16-23)	<.001	12.5	4.5-34.6	
Chen et al. (2018)	2570 pts Setting: nationwide	Retrospective record review<br><br>Cox Proportional Hazard Model	2570 (2.1%), 53 Stage not specified (ICD-9-CM codes 707.0)	12 (4)		HR		High
				ALS	<.001	8.82	4.9-15.9	
				Age	<.001	1.06	1.03-1.09	
				Stroke	<.05	2.3	1.05-5.07	
				Spinal cord injury	<.05	4.1	1.23-13.7	
Compton et al. (2008)	713 pts. Setting: ICU	Retrospective record review<br><br>Logistic regression	698 (17%), 121 Stage ≥ 2	32 (6)		OR		Moderate
				Male gender	.014	1.81	NR	
				Moist skin	.001	2.35	NR	
				Edematous skin	.002	2.25	NR	
				Centralized circulation	.001	2.4	NR	
				Mottled skin	.016	2.02	NR	
				Reddened skin	.001	2.31	NR	
Cox (2011)	347 pts. Setting: ICU	Retrospective record review<br><br>Logistic regression	347<br><br>Model 1: 347 (18.7%), 65 Stage ≥ 1<br><br>Model 2: 327 (13.8%), 45 Stage ≥ 2	*Model 1:* 15 (4)		OR		Low
				Age	.03	1.03	1.01-1.06	
				Hours in ICU	<.001	1.01	1.01-.1.01	
				Cardiovascular disease	.007	2.95	1.3-6.4	
				Braden scale mobility	.04	0.44	0.21-0.95	
				Model 2: 15 (4)				
				Hours in ICU	<.001	1.01	1.01-1.01	
				Cardiovascular disease	.02	3.38	1.22-9.35	
				Braden scale friction/shear	.01	5.72	1.42-22.95	
				Norepinephrine	.04	1.02	1.01-1.03	
Cox et al. (2020)	1460 pts. Setting: ICU	Retrospective Record Review<br><br>Logistic regression	1460 (25%), 365 Stage ≥ 1	13 (12)		OR		Low
				Female	.02	0.71	0.54-0.94	
				Age	<.001	1.02	1.01-1.03	
				Cardiovascular disease	.03	1.50	1.04-2.16	
				Peripheral vascular disease	<.001	2.30	1.43-3.71	
				Hypotension	<.001	1.90	1.37-2.65	
				Moderate to severe malnutrition	<.001	2.08	1.43-3.03	
				Septic shock	<.001	1.75	1.29-2.38	
				Pneumonia or influenza	.004	1.58	1.16-2.15	
				Cardiovascular surgery	<.001	2.53	1.54-4.13	
				Hemodialysis	.01	1.66	1.12-2.46	
				Norepinephrine	<.001	3.02	2.16-4.22	
				Braden scale score	<.001	0.84	0.79-0.89	

Defloor and Grypdonck (2005)	1772 pts.<br>Setting: long-term care facilities	RCT<br><br>Logistic regression	1458<br><br>Model 1: 1458 (20.7%), 302 Stage ≥ 1<br><br>Model 2: 1458 (11.7%), 171 Stage ≥ 2	*Model 1:* 19 (3)<br><br>Braden scale sensory perception<br>Skin condition<br>Existing PIs<br><br>*Model 2:* 19 (4)<br><br>Braden scale activity<br>Braden scale sensory perception<br>Skin condition<br>Existing PIs	 <br>.02<br><.001<br><.001<br><br><br><br>.03<br>.02<br><.001<br>.01	OR<br><br>0.76<br>1.49<br>2.25<br><br><br><br>0.67<br>0.73<br>1.63<br>1.85	 <br>0.61-0.96<br>1.21-1.85<br>1.43-3.54<br><br><br><br>0.47-0.96<br>0.55-0.95<br>1.25-2.12<br>1.13-3.04	Low
de Laat et al. (2007)	399 pts.<br>Setting: ICU	Prospective cohort study<br><br>Cox proportional hazard model	399 (35.1%), 140<br>Stage ≥ 2	11 (3)<br><br>Preventive transfers to specific mattresses<br>Shock or resuscitation<br>Friction and shear present	 <br><.001<br><br><.001<br>.02	HR<br><br>0.22<br><br>1.5<br>1.3	 <br>NR<br><br>NR<br>NR	Moderate
Donnelly et al. (2011)	239 pts<br>Setting: fracture trauma unit	RCT<br><br>Cox proportional hazard model	239 (16.3%), 39<br>Stage ≥ 1	NR (1)<br><br>Treatment group	 <br>NR	HR<br><br>0.21	 <br>0.08-0.54	Moderate
Dukes et al. (2018)	282 pts<br>Setting: patients overseen by the Critical Care Air Transport Team	Retrospective record review<br><br>Logistic regression	205<br>Stage not specified	16 (2)<br><br>BMI<br>Multiple transports (≥ 2 vs. 1)	 <br><.001<br><.001	OR<br><br>1.16<br>27.62	 <br>1.04-1.29<br>10.24-74.48	High
Ek (1987)	515 pts.<br>Setting: long-term medical ward	Prospective cohort study<br><br>Logistic regression	515 (7.6%), 39<br>Stage ≥ 1 equivalent<br><br>Model 1: baseline measure<br><br>Model 2: variables on day of PI or if PI free on 4th week of care	Model 1: 10 (1)<br><br>Norton scale mobility<br><br>Model 2: 10 (2)<br>General physical condition<br>Norton scale activity	 <br><.05<br><br><br><.01<br><.01	OR<br><br>NR<br><br><br>NR<br>NR	 <br>NR<br><br><br>NR<br>NR	High
Ek et al. (1991)	501 pts.<br>Setting: long-term medical ward	RCT<br><br>Multiple regression	495 (10.1%), 51<br>Stage ≥ 1 equivalent	NR (4)<br><br>Albumin<br>Norton scale mobility<br>Norton scale activity<br>Food intake	 <br><.001<br><.001<br><.05<br><.05	 <br>NR<br>NR<br>NR<br>NR	 <br>NR<br>NR<br>NR<br>NR	High

Forni et al. (2018)	359 pts Setting: university orthopedic hospital	RCT	359 (10%), 36 Stage ≥ 1	Model 1: 2 (2)		OR		Moderate
		Logistic regression	Model 1: logistic regression	Male gender	.035	2.28	1.06-4.91	
				Control vs intervention	.001	3.84	1.69-8.72	
		Cox proportional hazard model	Model 2: cox regression	Model 2: NR (1)		HR		
				Control vs intervention	.001	4.4	1.8-10.6	
Galivanche et al. (2020)	8871 pts Setting: multiple hospitals	Retrospective record review	8871 (5.2%), 457 Stage ≥ 1	Model 1: 17 (4)		OR		Low
				Preoperative sepsis	.001	4.04	1.14-14.37	
		Logistic regression	Model 1: preoperative comorbidities	High preoperative platelet count (>417)	.001	2.78	1.14-6.73	
				Insulin-dependent diabetes	<.001	2.32	1.39-3.86	
			Model 2: postoperative complications	Preoperative PI	<.001	2.26	1.23-4.17	
				Model 2: 12 (4)				
				Postoperative sepsis	.001	2.63	1.15-6.00	
				Postoperative pneumonia	<.001	2.47	1.42-4.28	
				Urinary tract infection	.001	2.04	1.12-3.71	
				Postoperative delirium	<.001	2.03	1.45-2.84	
Gao et al. (2018)	1963 pts (surgical) Setting: acute care hospital	Prospective cohort study	1940 (2.5%), 48 Stage ≥ 1	Model 1: 14 (6)		OR		Moderate
				Application of external force	.01	2.42	1.20-4.91	
		Logistic regression	Model 1: sig. clinical features	BMI classification (vs. marasmus <18.5)				
				Normal (18.5-22)	.03	0.34	0.14-0.83	
			Model 2: sig. clinical features with BMI and operation time dichotomized	Overweight (23-24)	.04	0.21	0.07-0.65	
				Operation time (h)	<.001	1.96	1.70-2.26	
				Operative position (vs. horizontal)				
				Prone position	.01	3.10	1.36-7.06	
				Cardiopulmonary bypass	<.001	6.89	2.66-17.83	
				Model 2: 12 (6)				
				Application of external force	.002	3.02	1.49-6.10	
				BMI < 18.5 (vs. ≥ 18.5)	.01	2.95	1.26-6.88	
				Operation time ≥ 6h (vs. < 6h)	<.001	14.30	6.39-32.01	
				Cardiopulmonary bypass	<.001	5.59	2.33-13.44	
				Intraoperative blood loss	.001	1.95	1.30-2.92	
González-Méndez et al. (2018)	335 pts Setting: ICU	Prospective cohort study	335 (8.1%), 27 Stage ≥ 1	6 (3)		HR		Moderate
				Severity (SAPS 3)	.009	1.04	1.01-1.07	

		Cox proportional hazard model		Days of immobilization	<.001	0.44	0.3-0.65	
				Complications	.01	4.89	1.45-16.41	
Goodridge et al. (1998)	330 pts<br>Setting: acute care hospital	Prospective cohort study<br><br>Logistic regression	330 (9.7%), 32<br>Stage ≥ 1	5 (1)		OR		High
				No. of prevention strategies used prior to PI appearance	<.001	1.35	NR	
Halfens et al. (2000)	320 pts.<br>Setting: hospital wards	Prospective cohort study<br><br>Logistic regression	320 (14.7%), 47<br>Stage ≥ 1	7 (4)		OR		Moderate
				Braden scale sensory perception	<.01	3.65	1.43-9.33	
				Age	<.01	2.33	1.39-3.92	
				Braden scale friction and shear	<.01	2.32	1.36-3.96	
				Moisture	<.01	2.08	1.23-3.51	
Han et al. (2018)	34.287 pts<br>Setting: university hospital	Retrospective record review<br><br>Logistic regression	34.287 (4%), 1368<br>Stage ≥ 1	10 (7)		OR		Low
				Female	<.001	1.36	1.21-1.54	
				Age	<.001	1.04	1.03-1.05	
				**Admission method**: Wheelchair	<.001	0.39	0.32-0.46	
				**Consciousness status:**				
				Drowsy	<.001	1.83	1.83-7.77	
				Stupor	.001	1.71	1.71-7.45	
				Semi-coma/coma	.02	1.17	1.17-5.19	
				Braden score	<.001	0.69	0.69-0.72	
Kim et al. (2019)	53.923 pts<br>Setting: tertiary and general hospitals	Retrospective record review<br><br>Cox proportional hazard model	53.923 (0.7%), 401<br>Stage ≥ 1	22 (11)		HR		Low
				Male gender	<.001	1.54	1.26-1.88	
				Age	.017	1.00	1.00-1.01	
				Fall	<.001	18.94	9.69-37.02	
				**Nursing needs:**				
				SpO_2 Monitoring	<.001	1.98	1.53-2.57	
				**ADL dependence:**				
				Position				
				Partial	<.001	3.37	2.27-5.01	
				Total	<.001	5.84	3.63-9.40	
				Ambulating				
				Partial	.049	0.66	0.44-1.00	
				Toilet use				
				Partial	.012	1.73	1.13-2.65	
				Total	<.001	3.25	2.05-5.14	
				**Staffing:**				
				RN-HPPD	<.001	0.20	0.13-0.30	
				NA-HPPD	<.001	0.12	0.04-0.34	

Lannering et al. (2016)	331 pts<br>Setting: nursing homes	Prospective cohort study<br><br>Cox proportional hazard model	331 (7.9%), 26<br>Stage ≥ 1<br><br>Model 1: n=323<br>Sociodemographics + DFRI scales<br><br>Model 2: n=328<br>Sociodemographics + MNA-SF scales<br><br>Model 3: n=330<br>Sociodemographics + MNS scales<br><br>Model 4:<br>Sociodemographics + total score of three scales	Model 1: 15 (4)		HR		High
				No. of drugs	<.05	1.16	1.01-1.34	
				No. of diagnoses	<.05	1.32	1.00-1.73	
				Hearing impairment scale	<.05	2.06	1.10-3.83	
				Hemipharesis/Limb impairment scale	<.05	1.96	1.04-3.70	
				Model 2: 10 (3)				
				Age	<.05	1.05	1.00-1.10	
				No. of drugs	<.05	1.14	1.03-1.27	
				Mobility scale	<.001	0.46	0.32-0.64	
				Model 3: 11 (4)				
				Age	<.05	1.06	1.01-1.11	
				No. of drugs	<.05	1.14	1.02-1.26	
				Mobility scale	<.05	0.56	0.33-0.93	
				Food intake scale	<.05	1.54	1.06-2.23	
				Model 4: 7 (3)				
				Age	<.05	1.05	1.00-1.10	
				No. of drugs	<.05	1.15	1.03-1.30	
				MNS	<.01	0.86	0.78-0.94	
Latimer et al. (2019)	1047 pts<br>Setting: general medical and surgical clinical units	Prospective cohort study<br><br>Logistic regression	1047 (10.8%), 113<br>Stage ≥ 1	3 (3)		OR		Low
				Age	<.001	1.05	1.02-1.08	
				No. of comorbidities	.002	1.22	1.08-1.37	
				Place of residence: aged care (vs. community)	<.001	1.75	1.32-2.32	
Magny et al. (2017)	585 pts<br>Setting: perioperative geriatric unit and tertiary teaching hospital	Prospective cohort study<br><br>Logistic regression	548 (12%), 63<br>Stage ≥ 1	32 (4)		OR		High
				Albumin (1g/L increase)	.003	0.90	0.84-0.96	
				Atrial fibrillation	.033	1.91	1.05-3.46	
				Chronic coronary artery disease	.014	2.16	1.17-3.99	
				Diabetes	.02	2.33	1.14-4.75	
Manzano et al. (2013)	221 pts<br>Setting: ICU	Prospective cohort study<br><br>Cox proportional hazard model	221 (19%), 42<br>Stage ≥ 2	4 (2)		HR		Low
				Age	<.001	1.05	1.02-1.09	
				Mattress group	.029	0.44	0.21-0.92	

Manzano et al. (2014)	330 pts<br>Setting: ICU	RCT<br><br>Cox proportional hazard model	329 (11.9%), 39<br>Stage ≥ 2	8 (2)		HR		Moderate
				Implementation rate of turning schedule (%)	<.001	0.97	0.95-0.98	
				Type of admission (surgical)	.02	2.44	1.14-5.23	
Milićević et al. (2012)	453 pts<br>Setting: rehabilitation clinic	Retrospective record review<br><br>Logistic regression	453 (15.5%), 70<br>Stage not specified	5 (2)		OR		Moderate
				PI on admission	<.001	4.10	1.90-8.70	
				FIM score on admission	.02	0.90	0.94-0.99	
Miller et al. (2020)	57227 pts<br>Setting: tertiary academic medical center	Retrospective record review<br><br>Logistic regression	30005 (0.8%), 241<br>Stage ≥ 1 equivalent PI	31 (7)		OR		Moderate
				Length of stay (days)	<.001	1.01	1.01-1.01	
				Mean hemoglobin	.03	0.89	0.81-0.99	
				Hba1c (>7%)	.02	1.72	1.08-2.74	
				Overweight (BMI > 35)	.04	0.60	0.38-0.97	
				Charlson comorbidity score	<.001	0.81	0.71-0.91	
				Minimum nutrition subscale	<.001	0.49	0.33-0.73	
				Minimum friction subscale	<.001	0.23	0.14-0.36	
Nixon et al. (2006)	1972 pts<br>Setting: acute care hospital	RCT<br><br>Logistic regression	1971 (10.5%), 207<br>Stage ≥ 2	13 (7)		OR		Low
				Hospital	.02	NR	NR	
				Acute admission	<.001	3.65	2.27-5.85	
				Baseline wound	<.001	2.96	1.73-5.08	
				Baseline skin trauma	.05	1.67	1.00-2.80	
				Baseline stage 1 PI	.001	1.95	1.31-2.91	
				Age	.03	1.02	1.002-1.04	
				Diabetes	.047	1.61	1.01-2.56	
O'Brien et al. (2014)	2695 pts<br>Setting: ICU	Retrospective record review	2548 (10.7%), 288<br>Stage ≥ 2	11 (7)		OR		Low
				Existing airway	<.001	5.28	3.63-7.67	
				BMI underweight (vs. normal)	.002	2.70	1.45-5.04	
				Noncardiac surgery	<.001	1.84	1.31-2.59	
				History of congestive heart failure	.001	1.78	1.27-2.49	
				History of renal failure	.001	1.75	1.27-2.39	
				ASA classification 4,5 (vs. 1,2,3)	.003	1.63	1.19-2.23	
				Age	<.001	1.02	1.01-1.03	
Okuwa et al. (2006)	259 pts<br>Setting: long-term nursing care/nursing home	Prospective cohort study<br><br>Cox proportional hazard model	259 (12.7%), 33<br>Stage ≥ 2	5 (3)		HR		Moderate
				Ankle brachial index	<.001	0.075	0.023-0.242	
				Male gender	.003	2.95	1.45-6.01	
				Length of bedfast period	.001	1.01	1.004-1.015	

Ooi et al. (1999)	5518 pts Setting: nursing homes	Retrospective record review<br><br>Logistic regression	5518 (11.4%), 629 Stage ≥ 2	7 (6)		OR		Low
				Facility effects				
				Intermediate risk	<.001	1.59	1.25-2.00	
				High risk	<.001	1.88	1.49-2.36	
				Age	.008	1.02	1.00-1.03	
				Diabetes	.01	1.35	1.07-1.69	
				Fecal/urinary incontinence	<.001	1.56	1.24-1.96	
				Transfers	<.001	1.45	1.17-1.79	
Osis and Diccini (2020)	240 pts Setting: neurotrauma reference hospital	Prospective cohort study<br><br>Logistic regression	240 (18.8%), 45 Stage ≥ 1	NR (4)		OR		Moderate
				Moderate TBI	<.01	7.03	1.86-26.47	
				Severe TBI	<.01	10.91	2.70-44.08	
				Noradrenaline use	<.01	1.04	1.01-1.07	
				Age	.01	1.03	1.00-1.05	
Park and Park (2017)	202 pts Setting: acute care hospital	Retrospective record review<br><br>Multiple Stepwise Regression	202 (100%), 202 Stage not specified	33 (11)				High
				Nutrition consultation	<.001	NR	NR	
				Ventilator	<.001	NR	NR	
				Sedative-hypnotics	<.001	NR	NR	
				Anti-inflammatory	<.001	NR	NR	
				Diarrhea	<.001	NR	NR	
				Steroid	<.001	NR	NR	
				Renal Failure	.002	NR	NR	
				Gastrointestinal tube	.02	NR	NR	
				Anticoagulant	.01	NR	NR	
				Decreased consciousness	.03	NR	NR	
				Trachestomy tube	.04	NR	NR	
Perneger et al. (2002)	1190 pts Setting: acute care hospital	Prospective cohort study<br><br>Cox proportional hazard model	1190 (10.8%), 129 Stage ≥ 1	13 (6)		HR		Low
				Age (vs 16-59)				
				70-79	NR	2.5	1.5-4.4	
				80-90	NR	3.8	2.3-6.4	
				90-96	NR	5.2	2.6-10.6	
				Braden/Norton mobility scale	<.001	1.4	1.1-1.8	
				Braden friction/shear scale	.03	1.5	1.0-2.2	
Popow et al. (2019)	733 pts Setting: hospital wards	Prospective cohort study<br><br>Logistic regression	733 (13%), 95 Stage not specified	20 (4)		OR		High
				Limited condition	.001	NR	NR	
				Oedema	.02	NR	NR	
				Neurological diagnosis	<.001	NR	NR	
				Higher protein concentration during hospitalization	.04	NR	NR	

Rademakers et al. (2007)	722 pts Setting: acute care hospital	Retrospective record review<br>Logistic regression	722 (29.6%), 214 Stage ≥ 2	11 (5)		OR		Low
				Diabetes	.02	1.70	1.08-2.66	
				Urinary tract infection	.004	1.87	1.22-2.85	
				Postoperative hip dislocation	.009	2.67	1.28-5.59	
				ASA class 3/4	.001	4.19	2.88-6.10	
				Time to surgery > 12h	.008	1.75	1.15-2.64	
Reed et al. (2003)	2771 pts Setting: chronic care hospital	Retrospective record review<br>Logistic regression	2771 (14.7%), 406 Stage ≥ 2	7 (6)		OR		Low
				PI stage 1	<.001	3.13	2.41-4.06	
				Malnourished	<.001	1.69	1.31-2.19	
				Urinary catheter on admission	<.001	1.55	1.38-1.75	
				DNR order	<.001	1.52	1.22-1.90	
				Confusion	.001	1.45	1.16-1.80	
				Low albumin level	.014	1.40	1.07-1.83	
Rondinelli et al. (2018)	466609 pts Setting: community hospital	Retrospective record review<br>Cox proportional hazard model	453050 (0.4%), 1661 Stage ≥ 1	12 (6)		HR		Low
				Age	<.001	1.36	1.25-1.45	
				COPS 2 Score	<.001	1.10	1.04-1.16	
				LAPS 2 Score	<.001	1.38	1.28-1.50	
				Braden score	<.001	0.63	0.59-0.67	
				Female gender	<.001	0.77	0.70-0.85	
				Admission type<br>ED/medical (yes)	<.001	0.62	0.52-0.74	
Sala et al. (2020)	1587 pts Setting: ICU	Retrospective record review<br>Logistic regression	1587 (5.1%), 81 Stage ≥ 1	6 (2)		OR		Low
				Mean arterial pressure < 60	NR	8.22	1.74-147.07	
				Lowest total Braden score	NR	0.79	0.70-0.88	
Salzberg et al. (1999)	226 pts Setting: acute care hospital	Retrospective record review<br>Logistic regression<br>Cox proportional hazard model	226 (38.5%), 87 Stage ≥ 1<br>Model 1: logistic regression<br>Model 2: Cox proportional hazard model	Model 1: 8 (3)		OR		High
				Extent of paralysis	<.001	NR	NR	
				Moisture	.003	NR	NR	
				Serum creatinine	.006	NR	NR	
				Model 2: 8 (8)		HR		
				Extent of paralysis	<.001	NR	NR	
				Moisture	<.001	NR	NR	
				Serum creatinine	.007	NR	NR	
				Incontinence	<.001	NR	NR	
				Albumin	.03	NR	NR	
				Mobility	.002	NR	NR	
				Pulmonary disease	.01			

				Level of activity	.04	NR<br>NR	NR<br>NR	
Sardo et al. (2018)	6552 pts<br>Setting: medical and surgical wards	Retrospective record review	6552<br>Stage ≥ 1	Model 1: 5 (2)		HR		Low
				Braden Mobility	<.001	2.46	2.00-3.03	
		Cox proportional hazard model	Model 1:<br>6552 (2.3%), 153	Braden Activity	<.05	1.24	1.02-1.52	
				Model 2: 5 (2)				
			Model 2:<br>1774 (5,5%), 97<br>Braden Score ≤ 16	Braden Mobility	<.01	1.71	1.21-2.41	
				Braden Activity	.05	1.63	1.00-2.65	
			Model 3:<br>4778 (1.2%), 56<br>Braden Score ≥ 16	Model 3: 5 (1)				
				Braden Mobility	<.001	3.20	2.03-5.04	
Schoonhoven et al. (2002)	208 pts<br>Setting: acute care hospital	Prospective cohort study	208 (10.1%), 21<br>Stage ≥ 2	12 (1)		OR		Moderate
		Logistic regression		Length of surgery	<.05	1.01	NR	
Schultz et al. (1999)	413 pts<br>Setting: acute care hospital	RCT	413 (21.5%), 89<br>Stage ≥ 1	7 (6)		OR		Low
		Logistic regression		Age	.005	1.05	1.03-1.08	
				Braden score on admission	.013	0.80	0.68-0.95	
				BMI	.015	0.91	0.85-0.98	
				Exhibits fear	.001	0.31	0.16-0.62	
				Diabetes	.013	2.52	1.21-5.25	
				Mattress overlay	.044	1.93	1.02-3.68	
Serpa et al. (2020)	1937 pts<br>Setting: acute care hospitals	Prospective cohort study	1937 (5.9%), 114<br>Stage not specified	18 (8)		OR		High
				Age	<.001	1.05	1.03-1.06	
		Poisson regression		Hypertension	.022	1.62	1.07-2.45	
				Public hospital (vs. private)	<.05	0.61	0.39-0.95	
				Length of hospital stay (days)	<.001	1.04	1.03-1.06	
				Clinical treatment (vs. surgical treatment)	.018	1.96	1.12-3.43	
				NRS 2002 (vs. no risk)				
				Moderate risk	.003	3.48	1.51-8.0	
				High risk	<.001	4.40	2.03-9.54	
				SGANS (vs. low risk)				
				Severe malnutrition	<.001	4.40	2.03-9.54	
Shaw et al. (2014)	297 pts (surgical)<br>Setting: medical center	Prospective cohort study	Model 1: 297 (9.8%), 29<br>Stage ≥ 1	Model 1: 10 (6)		OR		Moderate
				Age	NR	1.03	1.00-1.08	

		Logistic regression	PI assessment immediately after surgery in operating room	Type of anesthesia (general anesthesia yes vs. no)	NR	17.06	2.09-49.43	
				Type of operation position (non-supine vs. supine)	NR	32.26	4.48-48.79	
			Model 2: 297 (5.1%), 15 Stage ≥ 1	Type of surgery (orthopedics surgery vs general surgery)	NR	3.33	1.05-10.61	
				Admission Braden score	NR	0.95	0.91-0.99	
			PI assessment 30 min after surgery in post anesthesia recovery room	Number of nursing intervention	NR	0.94	0.90-0.98	
				Model 2: 10 (5)				
				Age	NR	1.06	1.00-1.12	
				Type of operation position (non-supine vs. supine)	NR	18.18	1.32-52.63	
				Type of surgery				
				orthopedics surgery vs general surgery	NR	9.29	1.05-28.50	
				cardiac surgery vs general surgery	NR	22.60	1.20-43.85	
				Numbers of nursing intervention	NR	0.95	0.91-0.99	
Sternal et al. (2016)	329 pts Setting: palliative care department	Retrospective record review	245 (15.9%), 39 Stage not specified	Model 1: NR (4)		OR		High
				Waterlow score	.001	1.14	1.06-1.23	
		Logistic regression	Model 1: factors at admission	Patients admitted from other wards	.007	2.94	1.34-6.45	
				Hemoglobin level	.012	0.81	0.69-0.96	
				Systolic blood pressure	.023	0.98	0.96-1.00	
			Model 2: factors during hospitalization	Model 2: NR (5)				
				Mean evening body temperature	.001	3.38	1.73-8.49	
				Waterlow score	<.001	1.19	1.09-1.31	
				Lowest recorded sodium concentration	.001	0.88	0.81-0.95	
				Mean systolic blood pressure	.003	0.96	0.93-0.98	
				Lowest recorded hemoglobin level	.016	0.80	0.67-0.96	
Strazzieri-Pulido et al. (2018)	766 pts Setting: ICU	Retrospective record review	766 (18.7%), 143 Stage ≥ 1	10 (5)		OR		Low
				Length of stay	<.001	1.09	1.06-1.12	
		Logistic regression		Mechanical ventilation	<.001	3.35	2.13-5.35	
				Palliative care	.005	6.69	1.94-31.14	
				Age	.003	1.02	1.01-1.03	
				NAS	.012	1.02	1.00-1.03	
Suriadi et al. (2008)	253 pts Setting: ICU	Prospective cohort study	253 (28.4%), 72 Stage ≥ 1	NR (3)		OR		Moderate
				Interface pressure	NR	2.2	1.6-2.9	
				Body temperature	NR	2.0	1.7-2.5	

				Cigarette smoking	NR	1.6	1.1-2.5	
Tescher et al. (2012)	12566 pts Setting: intensive or progressive care units	Retrospective record review<br>Cox proportional hazard model	12566 (3,3%), 416 Stage ≥ 2	15 (11)		HR[a]		Moderate
				Braden friction score (vs. score 3)				
				2	<.001	5.98	3.04-11.79	
				1	<.001	60.34	30.57-119.12	
				Braden activity score (vs. score 3/4)				
				2	<.001	1.79	1.26-2.56	
				Braden mobility score (vs. score 3/4)				
				2	.007	1.52	1.12-2.05	
				Braden sensory perception score (vs. score 4)				
				3	.004	1.62	1.16-2.26	
				2	.005	1.71	1.18-2.47	
				Braden moisture score (vs. score 4)				
				3	<.001	1.79	1.37-2.36	
				2	.004	1.77	1.20-2.61	
				Acure respiratory failure	.006	1.36	1.09-1.69	
				Surgery within 5 days	<.001	0.43	0.29-0.64	
				Surgery after 5 days	<.001	0.37	0.28-0.49	
Tourtual et al. (1997)	291 pts Setting: acute care hospital	Prospective cohort study<br>Logistic regression	291 (21.6%), 63 Stage ≥ 1	14 (2)		OR		Moderate
				Braden friction/shear	.01	NR	NR	
				Braden moisture	.007	NR	NR	
Tsaousi et al. (2015)	471 pts Setting: acute care hospital	Prospective cohort study<br>Logistic regression	471 (14.2%), 67 Stage ≥ 1	NR (3)		OR		Moderate
				Limited autonomy	<.001	6.46	3.21-12.97	
				MUST score	.001	3.83	1.73-8.46	
				Artificial diet	.02	1.87	1.28-2.80	
Tsaras et al. (2016)	236 pts Setting: ICU	Prospective cohort study<br>Logistic regression	210 (24.3%), 51 Stage ≥ 1	NR (4)		OR		Moderate
				Age	.03	1.04	1.01-1.07	
				Length of ICU stay (days)	<.001	1.17	1.11-1.23	
				Hemodialysis	.03	4.09	1.12-14.98	
				Hematocrit (%)	.04	0.91	0.82-0.99	
Vanderwe e et al. (2009)	235 pts Setting: nursing home	RCT<br>Cox proportional hazard model	235 (18.7%), 44 Stage ≥ 2	20 (5)		HR		Moderate
				Age (vs. < 80 yrs)				
				≥ 90 yrs	.015	0.35	0.15-0.82	
				Cerebral vascular accident	.042	1.94	1.10-3.70	
				Urinary incontinence	.004	0.24	0.09-0.64	
				Contractures	.04	2.02	1.03-3.95	
				Hypotension	.002	3.42	1.56-7.49	

Yatabe et al. (2013)	422 pts Setting: intermediate and acute care wards	Prospective cohort study	422 (7.1%), 30 Stage ≥ 2 equivalent	4 (2)		OR		Moderate
				Female	.01	0.33	0.14-0.77	
		Logistic regression		MNA	<.001	0.66	0.53-0.81	
Yoshimura et al. (2016)	298 pts Setting: acute care hospital	Retrospective record review	277 (10.8%), 30 Stage ≥ 1	3 (2)		OR		Moderate
				Perspiration	.037	3.09	1.07-8.58	
		Logistic regression		Length of surgery and core temperature: More than 6 hours and more than 38.1°C	<.001	8.45	3.04-27.46	
Zarei et al. (2019)	643 pts Setting: ICU	Retrospective record review	643 (8.9%), 57 Stage ≥ 1	8 (6)		OR		High
				Age ≥ 50 yrs (vs. < 50 yrs)	.001	2.9	1.48-5.67	
		Logistic regression		Smoking	.001	2.1	1.28-3.45	
				Length of stay ≥ 10 days (vs. < 10 days)	.001	4.0	2.27-7.05	
				Trauma hospitalization	.001	1.4	1.10-1.79	
				Diabetes	.001	3.5	1.52-8.03	
				Muscular paralysis	.001	5.1	2.84-9.15	

PI, pressure injury; NR, not reported; pts, Patients; RCT, randomized controlled trial; ICU, intensive care unit; [a] calculated effect size; SCI, spinal cord injury
