# Supplementary material for: Risk Factors for Pressure Injuries in Adult Patients: A Narrative Synthesis
Source: Int J Environ Res Public Health. 2022 Jan 11;19(2):761. doi: 10.3390/ijerph19020761 (PMC8776011; doi:10.3390/ijerph19020761)
Supplement: Supplementary file 1 [file ijerph-19-00761-s001.zip › S4 Risk factor domain _mechanical boundary condition_.pdf]

Supplementary Material S4: risk factor domain “mechanical boundary condition”

Table S2: Summary of evidence for risk factor domains and subdomains: “mechanical boundary condition”

**Variables**	**Studies with significant variables in multivariate model Overall RoB, Study author, variable name**		**Studies with non-significant variables in multivariate model Overall RoB, Study author, variable name**	
**Domain 1: mechanical boundary condition**	Stage ≥ 1 PI	Stage ≥ 2 PI	Stage ≥ 1 PI	Stage ≥ 2 PI
Body size<br>17 Studies<br>8/23 Variables (35%)	Low RoB<br>Schultz (1999) – body mass	Low RoB<br>O’Brien et al. (2014) – BMI <18.5	Low RoB<br>Defloor and Grypdonck (2005) – body mass	Low RoB<br>Brandeis (1994) – BMI<br>Defloor and Grypdonck (2005) – body mass
	Moderate RoB<br>Aloweni (2019) – BMI < 23<br>Miller (2020) – overweight (BMI > 35)<br>Gao (2018) – BMI < 18.5	Moderate RoB<br>Allman (1995) – decreased body weight (<58kg)	Moderate RoB<br>Bergstrom and Braden (1992) – ideal body weight (%)<br>Miller (2020) - underweight	Moderate RoB<br>Bergstrom and Braden (1992) – ideal body weight (%)<br>Compton (2008) – body weight (kg), height (cm), BMI<br>Vanderwee (2009) – underweight/overweight vs. Normal<br>Yatabe (2013) – BMI
	High RoB<br>Aghazadeh (2020) – weight (kg)<br>Dukes (2018) – BMI	High RoB	High RoB<br>Lannering (2016) – MNA-SF loss of weight, BMI<br>Park and Park (2017) – weight<br>Serpa (2020) – underweight/overweight/obese vs. eutrophic	High RoB
Friction/shear<br>11 Studies<br>7/14 Variables (50%)	Low RoB<br>Perneger (2002) – Braden friction/shear	Low RoB<br>Cox (2011) – Braden friction/shear	Low RoB<br>Cox (2011) – Braden friction/shear<br>Defloor and Grypdonck (2005) – Braden friction/shear<br>Sardo (2018) – Braden friction/shear	Low RoB<br>Defloor and Grypdonck (2005) – Braden friction/shear
	Moderate RoB<br>Halfens (2000) – Braden friction/shear<br>Miller (2020) – Minimum Braden friction score<br>Tourtual (1997) – Braden friction/shear	Moderate RoB<br>De Laat (2007) – friction and shear present<br>Tescher (2012) – Braden score 1, 2 vs. 3	Moderate RoB<br>Miller (2020) – mean Braden friction	Moderate RoB<br>Bergquist and Frantz (1999) – Braden friction/shear<br>Vanderwee (2009) – Braden friction/shear
	High RoB	High RoB	High RoB	High RoB
Interface pressure<br>16 Studies<br>10/19 Variables (53%)	Low RoB<br>Schultz (1999) – mattress overlay	Low RoB<br>Manzano (2013) - mattress	Low RoB<br>Defloor and Grypdonck (2005) – sitting posture<br>Kim (2019) – use of restraints	Low RoB<br>Defloor and Grypdonck (2005) – sitting posture<br>Nixon (2006) – overlay: mattress

			Strazzieri-Pulido (2018) – mattress type	
	Moderate RoB<br>Donnelly (2011) – heel support surface<br>Forni (2018) – foam dressing<br>Gao (2018) – application of external force during operation, prone position during operation<br>Shaw (2014) – nonsupine vs. supine position<br>Suriadi (2008) – S.S. scale interface pressure (≥ 35 mmHg, bony prominence, at sacrum)	Moderate RoB<br>De Laat (2007) – preventive transfer to specific mattress vs. no transfer	Moderate RoB	Moderate RoB<br>Anrys (2018) – static air mattress vs. alternating pressure air mattress<br>Schoonhoven (2002) – mattress on OR table (air and warming mattress), posture on OR table
	High RoB<br>Dukes (2018) – more than 2 transports	High RoB	High RoB<br>Dukes (2018) – vacuum spine board, en route time (h)	High RoB<br>Park and Park (2017) – restraint
Subdomain immobility<br>Mobility<br><br>28 Studies<br><br>26 /51 Variables (51%)	Low RoB<br>Cox (2011) – Braden mobility<br>Cox (2020) – cardiovascular surgery<br>Perneger (2002) – Braden mobility<br>Sardo (2018) – Braden mobility	Low RoB<br>Brandeis (1994) – ambulation difficulty<br>O'Brien et al. (2014) – noncardiac surgery<br>Rademakers (2007) – postoperative hip dislocation, time to surgery > 12h	Low RoB<br>Defloor and Grypdonck (2005) – spontaneous movements<br>Galivanche (2020) – pre-/postoperative use of mobility aid	Low RoB<br>Cox (2011) – Braden mobility<br>Defloor and Grypdonck (2005) – spontaneous movements<br>Rademakers (2007) – delayed commencement of mobilization (> 2days)
	Moderate RoB<br>Boyle and Green (2001) – coma/unresponsiveness/paralyzed and sedated<br>Gao (2018) – operation time<br>Shaw (2014) – use of general anesthesia (PI directly after surgery), orthopedics surgery, cardiac surgery (PI 30 min after surgery)<br>Yoshimura (2016) – > 6hrs surgery + core temperature > 38.1°C	Moderate RoB<br>Bergquist and Frantz (1999) – Braden mobility, recent fracture<br>Schoonhoven (2002) – length of surgery<br>Tescher (2012) – surgery, Braden score 2 vs. 3, 4<br>Vanderwee (2009) – contractures	Moderate RoB<br>Gao (2018) – preoperative waiting time (min)<br>Halfens (2000) – Braden mobility<br>Miller (2020) – time in OR<br>Shaw (2014) – use of general anesthesia (PI 30 min after surgery), neurosurgery, cardiac surgery<br>Tourtual (1997) – Braden mobility, limb weakness<br>Yoshimura (2016) – > 6hrs surgery + core temperature < 38.1°C	Moderate RoB<br>Bergquist and Frantz (1999) – ADL: toilet, transfer, bath<br>De Laat (2007) – major surgery<br>Schoonhoven (2002) – posture on OR table<br>Tescher (2012) – Braden score 1 vs. 3, 4<br>Vanderwee (2009) – Braden mobility
	High RoB<br>Bourdel Marchasson (2000) – lower limb fracture<br>Ek (1987) – Norton mobility<br>Ek (1991) – Norton mobility	High RoB	High RoB<br>Magny (2017) – delay to surgery<br>Park and Park (2017) – surgery<br>Popow (2019) – Norton mobility<br>Salzberg (1999) – Mobility	High RoB

	Lannering (2016) – MNA-SF mobility<br>Salzberg (1999) – extent of paralysis<br>Zarei (2019) – muscular paralysis			
Activity<br><br>29 Studies<br><br>20/46 Variables (43%)	<u>Low RoB</u><br>Han (2018) – admission with wheelchair<br>Kim (2019) – ADL: total and partial positioning, partial ambulating, total and partial toilet use<br>Sardo (2018) – Braden activity	<u>Low RoB</u><br>Defloor and Grypdonck (2005) – Braden activity<br>Ooi (1999) – transfer	<u>Low RoB</u><br>Defloor and Grypdonck (2005) – Braden activity<br>Han (2018) – admission with stretcher car<br>Kim (2019) – ADL: total ambulating, partial and total eating<br>Perneger (2002) – Braden activity	<u>Low RoB</u><br>Brandeis (1994) – ADL: bathing, transfer<br>Nixon (2006) – Braden activity bedfast vs walks frequently
	<u>Moderate RoB</u><br>González-Méndez (2018) – days of immobilisation<br>Tsaousi (2015) – limited autonomy in everyday activities	<u>Moderate RoB</u><br>Allman (1995) – immobility<br>Baumgarten (2004) – no. of ADL dependencies (0-6)<br>Bergquist and Frantz (1999) – limitation in activity to wheelchair, ADL dress (need assistance)<br>Okuwa (2006) – length of bedfast period<br>Tescher (2012) – Braden activity score 2 vs. 3, 4	<u>Moderate RoB</u><br>Gao (2018) – score daily activity ability<br>Halfens (2000) – Braden activity<br>Miller (2020) – mean, minimum Braden activity<br>Tourtual (1997) – Braden activity	<u>Moderate RoB</u><br>Baumgarten (2004) – bedfast<br>Bergquist and Frantz (1999) – Braden activity, ADL dress dependent<br>De Laat (2007) – inactivity, immobility<br>Tescher (2012) – Braden score 1 vs. 3, 4<br>Vanderwee (2009) – Braden activity
	<u>High RoB</u><br>Bourdel Marchasson (2000) – Kuntzmann score (1 point increase)<br>Ek (1991) – Norton activity<br>Popow (2019) – limited condition	<u>High RoB</u>	<u>High RoB</u><br>Dukes (2018)- duration of immobility (h)<br>Ek (1987) – Norton activity<br>Park and Park (2017) – activity disorder<br>Magny (2017) – ADL6, IADL4<br>Salzberg (1999) – level of activity	<u>High RoB</u>
Sensory perception<br><br>9 Studies<br><br>4/13 Variables (31%)	<u>Low RoB</u><br>Defloor and Grypdonck (2005) – Braden subscale	<u>Low RoB</u><br>Defloor and Grypdonck (2005) – Braden subscale	<u>Low RoB</u><br>Cox (2011) – Braden subscale<br>Perneger (2002) – Braden subscale<br>Sardo (2018) – Braden subscale	<u>Low RoB</u><br>Cox (2011) – Braden subscale
	<u>Moderate RoB</u><br>Halfens (2000) – Braden subscale	<u>Moderate RoB</u><br>Tescher (2012) – Braden score 2/3 vs. 4	<u>Moderate RoB</u><br>Miller (2020) – mean, minimum score<br>Tourtual (1997) – Braden subscale	<u>Moderate RoB</u><br>Tescher (2012) – Braden score 1 vs. 4<br>Vanderwee (2009) – Braden subscale
	<u>High RoB</u>	<u>High RoB</u>	<u>High RoB</u>	<u>High RoB</u>
Mental status<br><br>18 Studies<br><br>9/29 Variables (31%)	<u>Low RoB</u><br>Artico (2020) – ESASr score sleepy	<u>Low RoB</u><br>Reed (2003) - confused	<u>Low RoB</u><br>Defloor and Grypdonck (2005) – Norton mental state	<u>Low RoB</u><br>Brandeis (1994) - disorientation<br>Defloor and Grypdonck (2005) – Norton mental state

	Galivanche (2020) – postoperative delirium<br>Han (2018) – drowsy, stupor, semi-coma/coma<br>Schultz (1999) – exhibits fear		Galivanche (2020) – preoperative delirium, dementia<br>Han (2018) – confused<br>Perneger (2002) – Norton mental status<br>Sala (2020) – Glasgow Coma Scale ≤ 8	
	Moderate RoB	Moderate RoB	Moderate RoB	Moderate RoB<br>Anrys (2018) – neurological disorders<br>Baumgarten (2004) – dementia<br>Compton (2008) – min./max Glasgow Coma Scale<br>De Laat (2007) – limited response or unresponsive
	High RoB<br>Park and Park (2017) – decreased consciousness<br>Popow (2019) – neurological diagnosis	High RoB	High RoB<br>Ek (1987) – Norton mental state<br>Magny (2017) – dementia, hemiplegia/paraplegi a<br>Lannering (2016) – MNA-SF psychological stress, neuropsych impairment<br>Park and Park (2017) – cognitive impairment<br>Popow (2019) – Norton mental state	High RoB
Turning/repositioning	Low RoB	Low RoB	Low RoB	Low RoB
1 Study<br>1/2 Variables (50%)	Moderate RoB	Moderate RoB<br>Manzano (2014) – implementation rate of turning schedule	Moderate RoB	Moderate RoB<br>Manzano (2014) – 2h turning
	High RoB	High RoB	High RoB	High RoB

PI, pressure injury; RoB, risk of bias
