# Supplementary material for: Risk Factors for Pressure Injuries in Adult Patients: A Narrative Synthesis
Source: Int J Environ Res Public Health. 2022 Jan 11;19(2):761. doi: 10.3390/ijerph19020761 (PMC8776011; doi:10.3390/ijerph19020761)
Supplement: Supplementary file 1 [file ijerph-19-00761-s001.zip › S5 Risk factor domains _susceptibility and tolerance of the individual_.pdf]

Supplementary Material S5: risk factor domain "susceptibility and tolerance of the individual"

Table S3: Summary of evidence for risk factor domains and subdomains: "susceptibility and tolerance of the individual"

**Variables**	**Studies with significant variables in multivariate model Overall RoB, Study author, variable name**		**Studies with non-significant variables in multivariate model Overall RoB, Study author, variable name**	
**Domain 2: susceptibility and tolerance of the individual**	Stage ≥ 1 PI	Stage ≥ 2 PI	Stage ≥ 1 PI	Stage ≥ 2 PI
Age<br><br>41 Studies<br><br>25/42 Variables (60%)	Low RoB<br>Bergstrom (1996)<br>Cox (2011)<br>Cox (2020)<br>Han (2018)<br>Kim (2019)<br>Latimer (2019)<br>Perneger (2002)<br>Rondinelli (2018)<br>Strazzieri-Pulido (2018)<br>Schultz (1999)	Low RoB<br>Manzano (2013)<br>Nixon (2006)<br>O'Brien et al. (2014)<br>Ooi (1999)	Low RoB<br>Artico (2020)<br>Defloor and Grypdonck (2005)	Low RoB<br>Cox (2011)<br>Defloor and Grypdonck (2005)
	Moderate RoB<br>Aloweni (2019)<br>Halfens (2000)<br>Osis and Diccini (2020)<br>Shaw (2014)<br>Tsaras (2016)	Moderate RoB<br>Bergstrom and Braden (1992)<br>Vanderwee (2009)	Moderate RoB<br>Chan (2005)<br>González-Méndez (2018)<br>Miller (2020)<br>Tourtual (1997)	Moderate RoB<br>Allman (1995)<br>Baumgarten (2004) - ≥ 85y<br>Bergquist and Frantz (1999)<br>Compton (2008)<br>De Laat (2007)<br>Yatabe (2013)
	High RoB<br>Chen (2018)<br>Lannering (2016)<br>Serpa (2020)<br>Zarei (2019)	High RoB	High RoB<br>Dukes (2018)<br>Goodridge (1998)<br>Magny (2017)<br>Popow (2019)	High RoB
Sex<br><br>25 Studies<br><br>11/25 Variables (44%)	Low RoB<br>Cox (2020) – male<br>Han (2018) – female<br>Kim (2019) – male<br>Rondinelli (2018) – male	Low RoB<br>Brandeis (1994) – males in low incidence homes	Low RoB<br>Artico (2020) – male<br>Bergstrom (1996) – female	Low RoB
	Moderate RoB<br>Aloweni (2019) – female<br>Forni (2018) – male	Moderate RoB<br>Bergquist and Frantz (1999) – male<br>Compton (2008) – male<br>Okuwa (2006) – male<br>Yatabe (2013) – male	Moderate RoB<br>Chan (2005)<br>González-Méndez (2018) – male<br>Miller (2020) – male<br>Shaw (2014) – male	Moderate RoB<br>Baumgarten (2004) – male<br>Manzano (2014)
	High RoB	High RoB	High RoB<br>Chen (2018) - female<br>Dukes (2018) – male<br>Goodridge (1998)<br>Lannering (2016) – gender<br>Magny (2017)<br>Serpa (2020) – male	High RoB
Ethnic group	Low RoB<br>Bergstrom (1996) – white	Low RoB	Low RoB	Low RoB<br>Brandeis (1994) – white

6 Studies<br><br>2/6 Variables (33%)	Moderate RoB	Moderate RoB<br>Baumgarten (2004) – black	Moderate RoB<br>Aloweni (2019) – Chinese<br>Chan (2005)<br>Miller (2020) – white	Moderate RoB
	High RoB	High RoB	High RoB	High RoB
Laboratory values<br><br>21 Studies<br><br>17/36 Variables (47%)	Low RoB<br>Galivanche (2020) – high preoperative platelet count (>417)<br>Rondinelli (2018) – LAPS 2 score	Low RoB<br>Reed (2003) – low albumin level (<3 g/dl)	Low RoB	Low RoB<br>Nixon (2006) – hemoglobin on admission or preoperatively (g/l)
	Moderate RoB<br>Aloweni (2019) – anemia<br>Miller (2020) – Hba1c (>7%), mean hemoglobin<br>Tsaras (2016) - hematocrit	Moderate RoB<br>Allman (1995) – lymphopenia<br>Bergquist and Frantz (1999) – anemia	Moderate RoB<br>Bergstrom and Braden (1992) – albumin<br>Gao (2018) – preoperative hemoglobin<br>Miller (2020) – mean albumin	Moderate RoB<br>Bergstrom and Braden (1992) – albumin<br>Compton (2008) – max. creatinine, max. blood glucose, max. c-reactive protein, min. thromboplastin time, max. serum bilirubin<br>Manzano (2014) – albumin, anemia<br>Okuwa (2006) – sodium level, hemoglobin level
	High RoB<br>Bourdel Marchasson (2000) – 1 g/L albumin decrease<br>Ek (1991) – Albumin<br>Magny (2017) – 1 g/L albumin decrease<br>Popow (2019) – low protein concentration<br>Salzberg (1999) – serum creatinine<br>Sternal (2016) – lowest recorded sodium concentration, haemoglobin at admission, lowest recorded haemoglobin level	High RoB	High RoB<br>Chen (2018) – hyperlipidemia<br>Magny (2017) – creatinine, hemoglobin<br>Popow (2019) – high hemoglobin concentration<br>Salzberg (1999) - Albumin	High RoB
Nutrition<br><br>24 Studies<br><br>15/37 Variables (41%)	Low RoB<br>Cox (2020) – moderate to severe malnutrition (ICD 9 code)	Low RoB<br>Brandeis (1994) – difficulty feeding itself in high incidence homes<br>Reed (2003) – malnourished	Low RoB<br>Artico (2020) – ESASr score lack of appetite, artificial nutritio<br>Cox (2011) – Braden nutrition<br>Defloor and Grypdonck (2005) – Braden nutrition, extra nutrients (protein supplements), fluid intake<br>Nixon (2006) – Braden nutrition	Low RoB<br>Cox (2011) – Braden nutrition<br>Defloor and Grypdonck (2005) – Braden nutrition, extra nutrients (protein supplements), fluid intake

			Perneger (2002) – Braden dietary intake	
	Moderate RoB<br>Bergstrom and Braden (1992) – protein (%RDA), iron (%RDA)<br>Miller (2020) – Minimum Braden nutrition<br>Tsaousi (2015) – MUST score, artificial diet	Moderate RoB<br>Yatabe (2013) – MNA scale	Moderate RoB<br>Miller (2020) – mean Braden nutrition, nothing by mouth, malnutrition status	Moderate RoB<br>Anrys (2018) – Braden nutrition<br>Bergquist and Frantz (1999) – ADL dependent feed<br>Compton (2008) – parenteral nutrition<br>De Laat (2007) – inadequate food intake<br>Schoonhoven (2002) - malnutrition<br>Vanderwee (2009) – Braden nutrition
	High RoB<br>Bourdel Marchasson (2000) – control vs. nutritional intervention<br>Ek (1991) – Norton food intake<br>Park and Park (2017) – nutrition consultation, gastrointestinal tube<br>Serpa (2020) – moderate/high vs. without risk NRS 2002, severe malnutrition vs. low risk SGANS	High RoB	High RoB<br>Lannering (2016) – MNA-SF reduced food intake<br>Park and Park (2017) – nothing by mouth, malnutrition<br>Serpa (2020) – low vs. without risk NRS 2002, moderate malnutrition vs. low risk SGANS, adequate/probably inadequate/ completely inadequate vs. excellent Braden nutrition<br>Zarei (2019) – malnutrition	High RoB
Body temperature	Low RoB	Low RoB	Low RoB	Low RoB
7 Studies<br>3/7 Variables (43%)	Moderate RoB<br>Bergstrom and Braden (1992) – °F<br>Suriadi (2008) – S.S. scale body temperature (≥ 37.4°C)	Moderate RoB	Moderate RoB	Moderate RoB<br>Compton (2008)<br>Vanderwee (2009)
	High RoB<br>Sternal (2016) – mean evening body temperature during hospitalization	High RoB	High RoB<br>Ek (1987) – Norton body temperature<br>Popow (2019) – higher temperature	High RoB
Health status<br>29 Studies<br>15/52 Variables (29%)	Low RoB<br>Artico (2020) – ESASr score pain<br>Kim (2019) – previous falls<br>Latimer (2019) – no. of comorbidities<br>Strazzieri-Pulido (2018) – in palliative care<br>Rondinelli (2018) – COPS 2 score	Low RoB<br>Nixon (2006) – baseline wound<br>O'Brien et al. (2014) – ASA class 4/5<br>Rademakers (2007) – ASA class 3/4	Low RoB<br>Artico (2020) – KPS status >30 vs <30, ESASr score nausea/feeling sad/feeling nervous/lack of energy/overall wellbeing<br>Cox (2011) – APACHE<br>Defloor and Grypdonck (2005) – pain<br>Galivanche (2020) – preoperative SIRS/ bleeding disorder	Low RoB<br>Cox (2011) – APACHE<br>Defloor and Grypdonck (2005) – pain<br>Manzano (2013) – APACHE, SOFA score<br>Nixon (2006) – vascular patients vs acute care patients

			Han (2018) – pain<br>Strazzieri-Pulido (2018) – exit condition death	
	Moderate RoB<br>Aloweni (2019) – ASA grade ≥ 3<br>González-Méndez (2018) – Complications during stay, SAPS 3 severity<br>Milićević (2012) – FIM score<br>Miller (2020) – Charlson score	Moderate RoB<br>Anrys (2018) – pressure area related pain	Moderate RoB<br>Chan (2005) – secondary diagnosis, comorbidities<br>Gao (2018) – intraoperative blood loss<br>Milićević (2012) – ASIA scale completeness of lesion/level of injury, mode of trauma	Moderate RoB<br>Bergquist and Frantz (1999) – unable to cough, abnormal breath sounds<br>Compton (2008) – ICU mortality<br>Manzano (2013) – diarrhea<br>Manzano (2014) – APACHE
	High RoB<br>Park and Park (2017) – diarrhea	High RoB	High RoB<br>Dukes (2018) – injury type, injury cause<br>Lannering (2016) – no. of diagnoses<br>Magny (2017) – Charlson score, CIRS score<br>Park and Park (2017) – pain, hygiene management, weakness	High RoB
Diagnosis<br><br>13 Studies<br><br>8/24 Variables (33%)	Low RoB<br>Artico (2020) – cancer diagnosis vs other disease<br>Cox (2020) – influenza<br>Galivanche (2020) – urinary tract infection	Low RoB<br>O'Brien et al. (2014) – history of renal failure	Low RoB<br>Cox (2011) – infection<br>Galivanche (2020) – renal failure, ascites, cachexia (>10% weight loss in 6 mo. before surgery), preoperative wound infection, postoperative superficial infection, postoperative deep infection<br>Rondinelli (2018) – stroke	Low RoB<br>Cox (2011) – infection<br>O'Brien et al. (2014) – history of liver disease
	Moderate RoB<br>Osis and Diccini (2020) – moderate/severe TBI (GCS score)	Moderate RoB	Moderate RoB<br>Aloweni (2019) – renal disease	Moderate RoB
	High RoB<br>Chen (2018) – SCI, ALS, Stroke<br>Park and Park (2017) – renal failure	High RoB	High RoB<br>Magny (2017) – cancer, stroke<br>Park and Park (2017) – cancer<br>Popow (2019) – oncological diagnosis<br>Zarei (2019) – kidney failure	High RoB
Subdomain Skin/PI status<br>PI stage 1<br><br>4 Studies<br><br>4/4 Variables (100%)	Low RoB	Low RoB<br>Nixon (2006) – baseline PI grade 1b<br>Reed (2003) – PI stage 1	Low RoB	Low RoB
	Moderate RoB	Moderate RoB	Moderate RoB	Moderate RoB

		Allman (1995) – non-blanchable erythema of intact sacral skin<br>Anrys (2018) – non-blanchable erythema at baseline		
	High RoB	High RoB	High RoB	High RoB
Existing/previous PI<br><br>8 Studies<br><br>5/9 Variables (56%)	Low RoB<br>Defloor and Grypdonck (2005) – existing PI<br>Galivanche (2020) – preoperative PI	Low RoB<br>Defloor and Grypdonck (2005) – existing PI	Low RoB	Low RoB<br>Nixon (2006) – existing PI
	Moderate RoB<br>Milićević (2012) – existing PI	Moderate RoB<br>Baumgarten (2004) – PI on admission	Moderate RoB<br>Tourtual (1997) – admitted with PI	Moderate RoB<br>Allman (1995) – previous PI<br>Anrys (2018) – previous PI
	High RoB	High RoB	High RoB	High RoB
General skin status<br><br>7 Studies<br><br>11/17 Variables (68%)	Low RoB<br>Defloor and Grypdonck (2005) – skin condition (dry, macerated)	Low RoB<br>Defloor and Grypdonck (2005) – skin condition (dry, macerated)<br>Nixon (2006) – baseline skin trauma	Low RoB	Low RoB
	Moderate RoB	Moderate RoB<br>Allman (1995) – dry sacral skin<br>Compton (2008) – edematous, mottled, reddened skin, centralized circulation	Moderate RoB	Moderate RoB<br>Anrys (2018) – Dryness<br>Bergquist and Frantz (1999) - Edema<br>Compton (2008) – livid, hyperemic skin, cyanosis
	High RoB<br>Popow (2019) - Edema	High RoB	High RoB<br>Park and Park (2017) – Edema	High RoB
Moisture<br><br>14 Studies<br><br>8/20 Variables (40%)	Low RoB	Low RoB	Low RoB<br>Defloor and Grypdonck (2005) – Braden moisture scale<br>Perneger (2002) – Braden moisture scale<br>Sardo (2018) – Braden moisture scale	Low RoB<br>Defloor and Grypdonck (2005) – Braden moisture scale
	Moderate RoB<br>Halfens (2000) – moisture (urinary/fecal incontinence and sweat)<br>Tourtual (1997) – Braden moisture scale<br>Yoshimura (2016) – perspiration	Moderate RoB<br>Bergquist and Frantz (1999) – Skin drainage<br>Compton (2008) – moist skin<br>Tescher (2012) – Braden moisture scale score 2 vs. 4, score 3 vs. 4	Moderate RoB<br>Halfens (2000) – Braden moisture scale<br>Miller (2020) – mean, minimum Braden moisture scale	Moderate RoB<br>Anrys (2018) – Braden moisture scale<br>Bergquist and Frantz (1999) – Braden moisture scale<br>De Laat (2007) – constantly moist<br>Tescher (2012) – Braden moisture scale score 1 vs. 4

				Vanderwee (2009) – Braden moisture scale
	High RoB<br>Salzberg (1999) – moisture	High RoB	High RoB	High RoB
Incontinence<br><br>12 Studies<br><br>4/12 Variables (33%)	Low RoB	Low RoB<br>Brandeis (1994) – fecal incontinence in high incidence homes<br>Ooi (1999) – dual incontinence	Low RoB<br>Perneger (2002) – Norton incontinence	Low RoB<br>Brandeis (1994) – urinary incontinence<br>Reed (2003) – fecal incontinence
	Moderate RoB	Moderate RoB<br>Bergquist and Frantz (1999) – bowel/bladder incontinence<br>Vanderwee (2009) – urinary incontinence (decreases risk)	Moderate RoB<br>Tourtual (1997) – any incontinence	Moderate RoB<br>Allman (1995) – fecal incontinence<br>Baumgarten (2004) – urinary incontinence, fecal incontinence, dual incontinence<br>Bergquist and Frantz (1999) – urinary incontinence<br>Vanderwee (2009) – fecal incontinence, dual incontinence
	High RoB	High RoB	High RoB<br>Park and Park (2017) – incontinence<br>Popow (2019) – Norton incontinence<br>Salzberg (1999) – urinary incontinence	High RoB
Subdomain poor perfusion<br>Diagnosis related to oxygenation/circulation<br><br>28 Studies<br><br>27/59 Variables (46%)	Low RoB<br>Artico (2020) – ESASr score shortness of breath<br>Cox (2011) – cardiovascular disease<br>Cox (2020) – cardiovascular disease, peripheral vascular disease, septic shock, pneumonia<br>Galivanche (2020) – insulin-dependent diabetes, pre- and postoperative sepsis, postoperative pneumonia<br>Schultz (1999) – Diabetes	Low RoB<br>Brandeis (1994) – diabetes in high incidence home<br>Cox (2011) – cardiovascular disease<br>Nixon (2006) – Diabetes<br>O'Brien et al. (2014) – history of congestive heart failure<br>Ooi (1999) – Diabetes<br>Rademakers (2007) – Diabetes	Low RoB<br>Defloor and Grypdonck (2005) – skin circulation<br>Galivanche (2020) – non-insulin dependent diabetes, congestive heart failure, low preoperative platelet count, myocardial infarction, pulmonary embolism<br>Rondinelli (2018) – Diabetes	Low RoB<br>Defloor and Grypdonck (2005) – skin circulation<br>O'Brien et al. (2014) – history of diabetes
	Moderate RoB<br>Aloweni (2019) – respiratory disease<br>Boyle and Green (2001) – cardiovascular instability	Moderate RoB<br>Okuwa (2006) – ABI<br>Tescher (2012) – acute respiratory failure<br>Vanderwee (2009) – cerebral vascular accident	Moderate RoB<br>Aloweni (2019) – Diabetes, heart disease<br>González-Méndez (2018) – Diabetes<br>Miller (2020) – smoking history	Moderate RoB<br>Compton (2008) – sepsis<br>Vanderwee (2009) – Diabetes

	Suriadi (2008) –<br>S.S. scale<br>smoking		Tourtual (1997) –<br>diagnosed with CHF,<br>pulses	
	<u>High RoB</u><br>Magny (2017) –<br>Diabetes, chronic<br>coronary heart<br>disease<br>Zarei (2019) –<br>Diabetes, smoking	<u>High RoB</u>	<u>High RoB</u><br>Chen (2018) –<br>Diabetes, IHD,<br>Asthma, COPD<br>Magny (2017) –<br>periphal vascular<br>disease, heart valve<br>disease, cardiac<br>failure, COPD<br>Popow (2019) –<br>Diabetes,<br>arteriosclerosis,<br>vessel diagnosis,<br>COPD, Asthma<br>Salzberg (1999) –<br>pulmonary disease<br>Serpa (2020) –<br>Diabetes	<u>High RoB</u>
Oxygenation/ventilation<br><br>11 Studies<br><br>7/17 Variables (41%)	<u>Low RoB</u><br>Kim (2019) – SpO_2<br>monitoring<br>Strazzieri-Pulido<br>(2018) –<br>mechanical<br>ventilation	<u>Low RoB</u><br>O'Brien et al.<br>(2014) – existing<br>airway	<u>Low RoB</u><br>Rondinelli (2018) –<br>full code designation<br>Sala (2020) – FiO_2 ><br>50%	<u>Low RoB</u>
	<u>Moderate RoB</u><br>Gao (2018) –<br>cardiopulmonary<br>bypass (surgical)	<u>Moderate RoB</u><br>Bergquist and<br>Frantz (1999) –<br>oxygen use	<u>Moderate RoB</u><br>Shaw (2014) –<br>heart-lung machine<br>used	<u>Moderate RoB</u><br>Compton (2008) –<br>min. PaCO2, min.<br>arterial pH,<br>mechanical<br>ventilation
	<u>High RoB</u><br>Park and Park<br>(2017) –<br>ventilators,<br>tracheostomy tube	<u>High RoB</u>	<u>High RoB</u><br>Dukes (2018) –<br>mechanical<br>ventilation, lowest<br>SpO_2<br>Park and Park<br>(2017) –<br>endotracheal tube,<br>oxygen therapy	<u>High RoB</u>
Vasopressor<br><br>10 Studies<br><br>3/13 Variables (23%)	<u>Low RoB</u><br>Cox (2020) –<br>norepinephrine	<u>Low RoB</u><br>Cox (2011) -<br>norepinephrine	<u>Low RoB</u><br>Cox (2011) –<br>norepinephrine,<br>vasopressin<br>Sala (2020) –<br>vasopressor<br>administered<br>Strazzieri-Pulido<br>(2018) – vasoactive<br>drugs	<u>Low RoB</u><br>Cox (2011) –<br>vasopressin<br>O'Brien et al. (2014)<br>– use of<br>corticosteroid
	<u>Moderate RoB</u><br>Osis and Diccini<br>(2020) –<br>noradrenaline use	<u>Moderate RoB</u>	<u>Moderate RoB</u>	<u>Moderate RoB</u><br>Compton (2008) –<br>vasopressor<br>therapy<br>Schoonhoven<br>(2002) – method of<br>anesthesia (with or<br>without vasoactive<br>medication)
	<u>High RoB</u>	<u>High RoB</u>	<u>High RoB</u><br>Dukes (2018) –<br>vasoactive<br>medication<br>Park and Park<br>(2017) – cardiac<br>stimulants	<u>High RoB</u>

Blood pressure<br><br>14 Studies<br><br>10/24 Variables (42%)	Low RoB<br>Aloweni (2019) – Hypertension<br>Cox (2020) – Hypotension<br>Sala (2020) – mean arterial pressure < 60mmHg	Low RoB	Low RoB<br>Galivanche (2020) – Hypertension<br>Sala (2020) – fluid bolus volume	Low RoB<br>Cox (2011) – mean arterial pressure, systolic pressure, diastolic pressure
	Moderate RoB<br>Bergstrom and Braden (1992) – diastolic blood pressure	Moderate RoB<br>Bergstrom and Braden (1992) – systolic blood pressure<br>Vanderwee (2009) - Hypotension	Moderate RoB	Moderate RoB<br>Compton (2008) – max. heart rate, invasive monitoring<br>Vanderwee (2009) - Hypertension
	High RoB<br>Magny (2017) – atrial fibrillation<br>Serpa (2020) – Hypertension<br>Sternal (2016) – systolic blood pressure at admission, mean systolic blood pressure during hospitalization	High RoB	High RoB<br>Chen (2018) – Hypertension<br>Dukes (2018) – Highest systolic blood pressure, mean arterial pressure, average heart rate<br>Magny (2017) – Hypertension<br>Popow (2019) – Hypertension	High RoB

PI, pressure injury; RoB, risk of bias
