# Supplementary material for: Risk Factors for Pressure Injuries in Adult Patients: A Narrative Synthesis
Source: Int J Environ Res Public Health. 2022 Jan 11;19(2):761. doi: 10.3390/ijerph19020761 (PMC8776011; doi:10.3390/ijerph19020761)
Supplement: Supplementary file 1 [file ijerph-19-00761-s001.zip › S6 Additional domain _Diagnosis and Treatment_.pdf]

Supplementary Material S6: Additional domain “Diagnosis and Treatment”

Table S4: Summary of evidence for risk factor domains and subdomains: “Diagnosis and Treatment”

**Variables**	**Studies with significant variables in multivariate model Overall RoB, Study author, variable name**		**Studies with non-significant variables in multivariate model Overall RoB, Study author, variable name**	
**Diagnosis and Treatment**	Stage ≥ 1 PI	Stage ≥ 2 PI	Stage ≥ 1 PI	Stage ≥ 2 PI
Admission type<br>9 Studies<br>4/11 Variables (36%)	Low RoB<br>Rondinelli (2018) – ER admission with medical reason	Low RoB	Low RoB<br>Rondinelli (2018) – ER admission with surgical reason/with medical reason	Low RoB<br>Nixon (2006) – acute admission<br>O’Brien et al. (2014) – emergent admission
	Moderate RoB	Moderate RoB<br>Manzano (2014) – surgical admission	Moderate RoB<br>Miller (2020) – ICU admission	Moderate RoB<br>Schoonhoven (2002) – reason for admittance
	High RoB<br>Sternal (2016) – admission from other wards<br>Zarei (2019) – Trauma hospitalization	High RoB	High RoB<br>Popow (2019) – urgent admission	High RoB
Length of stay<br>14 Studies<br>8/15 Variables (53%)	Low RoB<br>Cox (2011) – ICU stay (h)<br>Strazzieri-Pulido (2018) – ICU stay	Low RoB<br>Cox (2011) – ICU stay (h)	Low RoB<br>Cox (2020) – ICU stay (d)	Low RoB
	Moderate RoB<br>Miller (2020) – days<br>Tsaras (2016) – ICU stay (days)	Moderate RoB	Moderate RoB<br>Aloweni (2019) – ICU stay, hospitalization stay (days)<br>Tourtual (1997)	Moderate RoB<br>Compton (2008) – ICU stay (days)<br>De Laat (2007) – length of ICU stay<br>Schoonhoven (2002) – number of days in ICU
	High RoB<br>Aghazadeh (2020) – days<br>Serpa (2020) – days<br>Zarei (2019) – days	High RoB	High RoB<br>Popow (2019) – long hospialization time	High RoB
Medication<br>8 Studies<br>5/12 (42%)	Low RoB	Low RoB	Low RoB<br>Kim (2019) – IV medication	Low RoB<br>Brandeis (1994) – antipsychotic drug use in high incidence homes
	Moderate RoB	Moderate RoB	Moderate RoB	Moderate RoB<br>Bergquist and Frantz (1999) – prescribed use of analgesics<br>Compton (2008) – insulin therapy, sedation<br>Schoonhoven (2002) – preoperative medication anticoagulants,

				beta-blockers, analgesics<br>Vanderwee (2009) – sleeping medication/tranquilizers
	High RoB<br>Lannering (2016) – no. of drugs<br>Park and Park (2017) – sedative hypnotics, anti-inflammatory drugs, steroids, anticoagulant	High RoB	High RoB<br>Park and Park (2017) – analgesic, anxiolytics	High RoB
Risk assessment<br><br>26 Studies<br><br>17/34 Variables (50%)	Low RoB<br>Artico (2020) – Braden scale at risk vs not at risk<br>Bergstrom (1996) – total Braden score<br>Cox (2020) – total Braden score<br>Han (2018) – total Braden score<br>Rondinelli (2018) – total Braden score<br>Sala (2020) – lowest total Braden score<br>Schultz (1999) – admission Braden scale score	Low RoB	Low RoB<br>Cox (2011) – total Braden score<br>Defloor and Grypdonck (2005) – Norton general physical condition<br>Perneger (2002) – Norton physical status	Low RoB<br>Cox (2011) – total Braden score<br>Defloor and Grypdonck (2005) – Norton general physical condition
	Moderate RoB<br>Aloweni (2019) – preoperative Braden score ≥ 14<br>Bergstrom and Braden (1992) – total Braden score<br>Chan (2005) – high and moderate risk vs. low risk Braden scale<br>Shaw (2014) – total Braden score for PI directly after surgery	Moderate RoB<br>Anrys (2018) – total Braden score<br>Bergstrom and Braden (1992) – total Braden score	Moderate RoB<br>Miller (2020) – mean/minimum Braden score<br>Shaw (2014) – total Braden score for PI 30 min after surgery<br>Tourtual (1997) – total Braden score	Moderate RoB<br>Bergquist and Frantz (1999) – total Braden score<br>Compton (2008) – Waterlow score
	High RoB<br>Aghazadeh (2020) – total Braden score<br>Bourdel-Marchasson (2000) – Norton score (5-10 vs. >14)<br>Sternal (2016) – Waterlow score at admission, mean Waterlow score during hospitalization	High RoB	High RoB<br>Ek (1987) – Norton scale: social activity, general physical condition<br>Goodridge (1998) – total Braden score<br>Park and Park (2017) – total Braden score<br>Popow (2019) – Norton physical state, Norton total score	High RoB
Nursing/treatment<br><br>14 Studies<br><br>13/21 Variables (62%)	Low RoB<br>Artico (2020) – urinary catheter<br>Cox (2020) – hemodialysis	Low RoB<br>Reed (2003) – urinary catheter on admission, DNR order	Low RoB<br>Galivanche (2020) – preoperative/intraoperative/postoperative RBC transfusion, failure to wean	Low RoB

	Kim (2019) – registered nurse, nurse assistant<br>Latimer (2019) – aged care facility<br>Strazzieri-Pulido (2018) – NAS score (increased labour)			
	Moderate RoB<br>Shaw (2014) – no. of nursing intervention<br>Tsaras (2016) - hemodialysis	Moderate RoB<br>Bergquist and Frantz (1999) – adult child as caregiver	Moderate RoB	Moderate RoB<br>Compton (2008) – TISS score, urinary catheter
	High RoB<br>Goodridge (1998) – no. of prevention strategies used prior to PI apearrance<br>Serpa (2020) – clinical vs. surgical treatment	High RoB	High RoB<br>Park and Park (2017) – foley catheter<br>Serpa (2020) – public vs. private hospital	High RoB

PI, pressure injury; RoB, risk of bias
